# [^18^F]FDG Accumulation in Early Coronary Atherosclerotic Lesions in Pigs

**DOI:** 10.1371/journal.pone.0131332

**Published:** 2015-06-29

**Authors:** Miikka Tarkia, Antti Saraste, Christoffer Stark, Tommi Vähäsilta, Timo Savunen, Marjatta Strandberg, Virva Saunavaara, Tuula Tolvanen, Jarmo Teuho, Mika Teräs, Olli Metsälä, Petteri Rinne, Ilkka Heinonen, Nina Savisto, Mikko Pietilä, Pekka Saukko, Anne Roivainen, Juhani Knuuti

**Affiliations:** 1 Turku PET Centre, University of Turku and Turku University Hospital, Turku, Finland; 2 Research Centre of Applied and Preventive Cardiovascular Medicine, University of Turku, Turku, Finland; 3 Heart Center, Turku University Hospital and University of Turku, Turku, Finland; 4 Department of Forensic Medicine, University of Turku, Turku, Finland; 5 Institute of Clinical Medicine, University of Turku, Turku, Finland; University Hospital Medical Centre, GERMANY

## Abstract

**Objective:**

Inflammation is an important contributor to atherosclerosis progression. A glucose analogue ^18^F-fluorodeoxyglucose ([^18^F]FDG) has been used to detect atherosclerotic inflammation. However, it is not known to what extent [^18^F]FDG is taken up in different stages of atherosclerosis. We aimed to study the uptake of [^18^F]FDG to various stages of coronary plaques in a pig model.

**Methods:**

First, diabetes was caused by streptozotocin injections (50 mg/kg for 3 days) in farm pigs (n = 10). After 6 months on high-fat diet, pigs underwent dual-gated cardiac PET/CT to measure [^18^F]FDG uptake in coronary arteries. Coronary segments (n = 33) were harvested for *ex vivo* measurement of radioactivity and autoradiography (ARG).

**Results:**

Intimal thickening was observed in 16 segments and atheroma type plaques in 10 segments. Compared with the normal vessel wall, ARG showed 1.7±0.7 times higher [^18^F]FDG accumulation in the intimal thickening and 4.1±2.3 times higher in the atheromas (*P* = 0.004 and *P* = 0.003, respectively). *Ex vivo* mean vessel-to-blood ratio was higher in segments with atheroma than those without atherosclerosis (2.6±1.2 vs. 1.3±0.7, *P* = 0.04). *In vivo* PET imaging showed the highest target-to-background ratio (TBR) of 2.7. However, maximum TBR was not significantly different in segments without atherosclerosis (1.1±0.5) and either intimal thickening (1.2±0.4, *P* = 1.0) or atheroma (1.6±0.6, *P* = 0.4).

**Conclusions:**

We found increased uptake of [^18^F]FDG in coronary atherosclerotic lesions in a pig model. However, uptake in these early stage lesions was not detectable with *in vivo* PET imaging. Further studies are needed to clarify whether visible [^18^F]FDG uptake in coronary arteries represents more advanced, highly inflamed plaques.

## Introduction

Inflammation is an important contributor to atherosclerosis progression and key feature of plaques at high risk of rupture [1]. A glucose analogue ^18^F-fluorodeoxyglucose ([^18^F]FDG) has been used as a tracer with positron emission tomography (PET) to non-invasively detect inflammation in atherosclerotic plaques [2,3]. The degree of [^18^F]FDG accumulation correlates with the amount of macrophages in atherosclerotic plaques [4,5].

Imaging of [^18^F]FDG uptake in the coronary arteries is challenging due to their small size and continuous movement during the respiratory and cardiac cycles. We have shown that PET signal from the coronary arteries can be enhanced by at least 86% by correcting the respiratory and cardiac motion by dual-gated PET acquisition [6,7]. Some clinical studies have already shown increased [^18^F]FDG uptake in coronary arteries [8–14]. The highest uptake of [^18^F]FDG has been shown in the culprit lesions of acute coronary syndromes [11], but the amount of [^18^F]FDG uptake in different stages of coronary atherosclerosis is unknown

Tissue samples cannot be obtained from human coronary arteries. [^18^F]FDG and *in vivo* molecular imaging studies of coronary artery disease are not feasible in small animal models due the small size of coronary arteries. Pig is a potential model for cardiovascular research because the size, anatomy and physiology of circulatory organs correspond closely to that in humans [15]. Although pigs are resistant to coronary atherosclerosis, formation of extensive coronary atherosclerosis has been observed in farm pigs after combination of streptozotozin induced diabetes and high-fat diet induced hypercholesterolemia [16,17].

We wanted to test how [^18^F]FDG is taken up by various stages of atherosclerotic coronary plaques and study the feasibility of imaging coronary atherosclerotic plaque inflammation by combination of dual-gated [^18^F]FDG PET and coronary computed tomography angiography (CTA) in a pig model of coronary artery disease induced by streptozotozin–induced diabetes and dietary hypercholesterolemia. In order to validate the *in vivo* imaging findings, we studied histology of coronary arteries and measured [^18^F]FDG uptake in the coronary arteries by gamma counter and digital autoradiography of tissue sections.

## Materials and Methods

### Experimental model of atherosclerosis

Seventeen Finnish landrace pigs (age 2–3 months, weight 21.3±1.4 kg, range 18.8–23.9 kg) underwent intervention to induce atherosclerosis. Age-matched healthy control animals obtained from pig farm (n = 7) were used as controls in some experiments.

In order to accelerate the formation of atherosclerotic plaques, diabetes was induced by destroying pancreatic beta cells as described previously [16]. Pigs were sedated with ketamine (30 mg/kg intramuscularly, Ketalar, Amgen Technology Ireland, Dublin, Ireland) and streptozotocin 50 mg/kg (Zanosar, Pharmacia & Upjohn, MI, USA) was injected via ear vein once a day for 3 days. Glucose was given 25 g *per os* twice daily for 2 days to offset insulin release from pancreas. Five days after the last streptozotocin injection, atherogenic diet was started. Of the seventeen animals, seven were excluded within the first weeks after streptozotozin treatment due to persistently low level of blood glucose despite streptozotozin treatment. In order to promote development of atherosclerotic lesions of different stages, pigs were given a diet supplemented with either 1.5% cholesterol and 15% lard (HF, n = 6) or 4% cholesterol, 20% lard and 1.5% sodium cholate (HF+c, n = 4) (Special Diet Services, Witham, UK) for 6 months. All pigs were housed and fed in individual pens under a 12-h light/12-h dark cycle. Water was provided ad libitum. Animal health was monitored daily.

Body weight was measured weekly. Blood glucose was measured by obtaining drop sample from ear vein at baseline and every fifth week throughout the study. Blood glucose was measured with a glucometer (One Touch, UltraEasy, LifeScan, Inc., Milpitas, CA, USA). Insulin was administered in one animal to maintain target glucose levels of <20 mmol/L. Plasma cholesterol was measured at baseline and at study weeks 10, 15 and 25. Blood sample for cholesterol measurements was taken from jugular vein into lithium heparin plasma tubes. Plasma was separated and cholesterol levels were analysed with Modular P800 automatic analyzer (Roche Diagnostics GmbH, Mannheim, Germany).

All animal experiments were made according to European Community Guidelines for the use of experimental animals and approved by Finnish National Animal Experiment Board.

### PET/CT imaging

After six months of high-fat diet, myocardial perfusion and coronary [^18^F]FDG uptake were studied by PET and computed tomography (CT) imaging in the atherosclerotic pigs. Myocardial perfusion was also studied in healthy control animals.

In order to suppress myocardial glucose uptake, animals were prepared with an overnight fast and carbohydrate free diet (ground meat) was given for two days before PET imaging [18]. Prior to imaging, animals were sedated with midazolam 1 mg/kg i.m. (Midazolam Hameln 5 mg/mL, Hameln pharmaceuticals GmbH, Hameln, Germany) and xylazine 4 mg/kg i.m. (Rompun vet 20 mg/ml, Bayer Animal Health GmbH, Leverkusen, Germany). Animals were intubated and connected to a volume-triggered respirator and ventilated with 40% oxygen at the frequency of 16 breaths/min (tidal volume 8–10 mL/kg). The internal carotid artery was cannulated for blood sampling and for measurement of blood pressure, and the external jugular vein was cannulated for PET tracer administration. The anaesthesia was maintained throughout the imaging studies with continuous i.v. infusion of propofol 10−50 mg/kg/h (Propofol Lipuro 20 mg/mL, B. Braun Melsungen AG, Melsungen, Germany).

PET studies were performed with a hybrid scanner containing PET and 64 slice CT (Discovery VCT, General Electric Medical Systems, Milwaukee, WI, USA) operated in 2-dimensional mode for perfusion studies and 3-dimensional for [^18^F]FDG studies.

All animals had myocardial perfusion PET study with [^15^O]water at rest and during vasodilator stress. Animals were imaged in the supine position. At the beginning, low-dose CT scan (30 mA, 80 kV) was performed for attenuation correction. [^15^O]water (Radiowater Generator, Hidex Oy, Finland) was injected i.v. as a slow bolus. The injected radioactivity was 1021±287 MBq (range 405–1640 MBq). Dynamic PET scan was started at the time of injection. The acquisition times were as follows: 14×5 s, 3×10 s, 3×20 s, 4×30 s (total duration 4 min 40 s. Approximately 10 min after the rest study, scan was repeated during i.v. adenosine (Adenosin Life Medical, Life Medical Sweden AB, Stocksund, Sweden) infusion at rate of 200 μg/min/kg starting 2 minutes prior to scan and continued until the end of scan.

The [^18^F]FDG PET consisted of listmode acquisition for 30 minutes starting 120 minutes after i.v. injection of 883±152 MBq (594–1032 MBq) of the tracer. For attenuation correction, low-dose CT with 120 kV and 80 mA and 4D CINE CT with 120 kV and 438 mA were performed.

Respiratory motion was recorded with a real-time position management (RPM) respiratory gating system (Varian Medical Systems, USA). The RPM system works in conjunction of PET, sending a respiratory trigger to the PET list-mode data at every peak inspiration. The system also allows retrospective respiratory gating of CINE CT in regard to the recorded respiratory signal. Additionally, the recorded respiratory signals during PET and CT can be exported for off-line processing. An IVY-3150 3-lead electrocardiography system (ECG) (IVY Biomedical Systems, USA) was used to obtain a cardiac trigger from each R-peak, which is temporally synchronized with the PET acquisition and saved to the PET list-mode data for cardiac gating.

Finally, coronary computed tomographic angiography (CTA) with iodinated contrast agent (Omnipaque 350 mg I/mL, Amersham Health AS, Nydalen, Oslo, Norway) was performed to visualize anatomy of coronary arteries according to a previously described protocol [19]. Contrast agent (100 mL) was administered at 4 mL/s via ear vein and flushed with 100 mL of physiological saline followed by a helical ECG gated scan. Tube voltage was 120 kV and current was 700 mA. Diastolic phase with the best image quality was selected for analysis.

### PET image reconstruction

The acquired list-mode data was divided into cardiac and respiratory gates and were combined as dual gates compensating for both respiratory and cardiac motion as described earlier [6,7]. According to our previous experiences, expiratory and diastolic phases were chosen for reconstruction of dual gated images. The dual gated PET images were reconstructed with an iterative reconstruction algorithm (3D-OSEM) with two iterations, 28 subsets and 6 mm Gaussian post-filtering. No resolution modelling was applied to the gated images. The device produces 47 axial planes with a slice thickness of 3.27 mm and a total transaxial field of view of 15.2 cm. Voxel size was 1.37×1.37×3.27 mm. The image matrix size was 256×256 with a 35 cm field of view (FOV). All quantitative corrections including attenuation, scatter, randoms, detector normalization and dead time were included in the reconstruction. Respiratory gated CINE CT images were used for attenuation correction of PET images, where the corresponding respiratory phase in PET was matched with a similar phase in CT.

### PET image analysis

Myocardial perfusion images were analysed with Carimas 2 software (Turku PET Centre, Turku, Finland; http://www.turkupetcentre.fi/carimas) as previously described [20]. The segmental myocardial blood flow (MBF) was quantified as ml/g/min and myocardial flow reserve (MFR) was calculated by dividing stress MBF with rest MBF.

[^18^F]FDG images were analysed using Carimas software fused with coronary CTA image as an anatomical reference. Co-registration of images was visually confirmed using [^18^F]FDG uptake in the aortic root, myocardium and bone marrow as anatomical landmarks. The length of histological sections was measured with ruler and corresponding length of each vessel was used in *in vivo* PET analysis. Separate regions-of-interest (ROI) were drawn covering proximal left anterior descending coronary artery including the left main coronary artery (proximal LAD), proximal left circumflex coronary artery (LCX) and proximal right coronary artery (RCA) and blood volume in descending aorta using CTA as a reference. In order to match *in vivo* imaging findings with histology, the length of the region of interest in each coronary segment was defined as centimetres from the ostium of the left or right coronary artery or bifurcation of the LCX in the *ex vivo* coronary artery sample. [^18^F]FDG uptake was expressed as peak target-to-background (TBR) ratio calculated by dividing the maximum standardized uptake value (SUV) of each coronary artery segment by the mean SUV value of blood that was measured from the descending aorta using a volume-of-interest (VOI) size of approximately 2 cm^3^.

### Tissue sampling

Animals were sacrificed by i.v. injection of potassium chloride (B. Braun Medical Oy, Helsinki, Finland) immediately after completing the PET/CT scan and the coronary arteries were prepared. Samples of the proximal LAD, proximal LCX and proximal RCA coronary arteries of approximately 3 to 5 cm long together with a blood sample were collected. Samples were weighed and radioactivity was measured using the gamma counter (1480 Wizard 3″; PerkinElmer/ Wallac, Turku, Finland). The radioactivity concentration was expressed as SUV = ([organ activity/organ weight]/[total given radioactivity/animal body weight]). In addition, vessel-to-blood ratios were calculated.

The coronary artery samples were mounted, frozen in isopentane mixed with dry ice, and cut longitudinally into serial 7 μm and 40 μm cryosections. Samples of 40 μm were immediately exposed overnight against a phosphor imaging plate (BAS-TR2025, Fuji Photo Film Co. Ltd., Tokyo, Japan). The distribution of radioactivity on the plate was visualized and quantified using Fluorescent Image Analyzer (Fujifilm FLA-5100, Fuji Photo Film Co. Ltd., Tokyo, Japan). The 40 μm cryosections were stained with hematoxylin & eosin (HE). The sections of 7 μm were stored at -70°C and stained with HE, Movat's pentachrome and macrophage staining (MAC387, ab22506, Abcam, Cambridge, MA, USA).

### Autoradiography analysis

Tissue sections stained with HE and Movat's pentachrome were visualized under a light microscope and digitally photographed. Autoradiographs and HE images of the 40 μm cryosections were co-registered and [^18^F]FDG accumulation was measured by drawing ROIs covering atherosclerotic lesion or adjacent normal or non-atherosclerotic vessel wall using TINA 2.10f software (Raytest Isotopenmessgeräte GmbH., Straubenhardt, Germany) as previously described [21,22]. The results were expressed as photostimulated luminescence per square millimetre (PSL/mm^2^) and lesion-to-normal vessel wall ratios for each segment were calculated by dividing average photostimulated luminescence results of ROIs in the intimal thickening or atheroma by that of ROIs in the normal vessel wall.

### Histological analysis

Histological coronary sections of 8 μm stained with Movat's pentachrome were analysed visually under the light microscope and graded with following scores: 1 = healthy normal vessel wall, 2 = intimal thickening, 3 = atheroma. Intimal thickening was defined as thickened intima without lipid core or fibrous cap. Atheroma was defined as a lesion with clearly defined lipid or necrotic core with various degrees of overlying connective tissue. Healthy segments were defined as lack of intimal thickening or atheroma in histological sections[23].

### Vascular reactivity analysis

Distal parts of the left anterior descending artery and the posterior descending right coronary artery were excised immediately after sacrifice, placed in ice-cold oxygenated Krebs solution and mounted in a wire-myograph system (Myo Technology A/S, Multi Myograph System, model 610M) for isometric tension recordings. Two different-sized vessel beds were used in the study; small and large arteries with an inner diameter of ~150 and ~300 μm, respectively. After equilibration and normalization of the vessel segments, the arteries were contracted repeatedly with 62 mM KCl until maximal and reproducible contractions were obtained. Arteries were precontracted with the tromboxane A2 mimetic U46619 and studied for endothelium-dependent and-independent relaxation responses to bradykinin and sodium nitroprusside, respectively. The contribution of vasodilator NO to bradykinin-evoked relaxation was determined by the inhibitory effect of a nitric oxide synthase inhibitor (Nω-nitro-L-arginine, L-NNA, 100 μM). Data was collected and analyzed using PowerLab and Chart5 softwares (ADI Instruments, Colorado Springs, CO, USA).

### Statistical analyses

Correlation between histological findings and plasma cholesterol content was calculated with Pearson correlation by using mean value for histological grade covering all coronary segments of each animal. Differences between classes defined by histological grading and *ex vivo* vascular responses were evaluated with one-way ANOVA and Bonferroni post hoc tests. Additionally, One-sample T Test was used to compare autoradiography results between healthy vessel wall and intimal thickening. Differences in myocardial blood flow measurements were evaluated with independent-samples T Test. Data are presented as means±SD. P-values <0.05 were considered significant.

## Results

The final study group consisted of 10 atherosclerotic pigs that all survived the 6 month diet intervention. Their characteristics after 25-week follow-up are shown in Table 1. Blood glucose varied between 6.9 and 20.0 mmol/L (12.3±4.7) and cholesterol level was between 6.4 and 23.6 mmol/L (12.7±5.1) at the end of the study. In healthy control animals, blood glucose and cholesterol levels were 6.1±2.5 mmol/L and 2.0±0.3 mmol/L, respectively. Body weight in the atherosclerotic animals was 127±8 kg and in control animals 82±10 kg.

**Table 1:** Characteristics of pigs with induced diabetes and atherosclerosis at the end of study.

Animal #	1	2	3	4	5	6	7	8	9	10
Diet	HF	HF	HF	HF	HF	HF	HF+c	HF+c	HF+c	HF+c
Body weight (kg)	130	111	130	115	135	130	130	130	130	125
Blood glucose (mmol/L)	20	16.6	17.2	9.1	6.9	14.1	9.1	7.5	8.2	14.5
Plasma total cholesterol (mmol/L)	10.8	6.4	7.2	9.8	12.9	13.6	23.6	15	17.1	10.8
HDL cholesterol (mmol/L)	2.09	2.07	2.69	2.97	3.21	2.94	5.72	5.77	5.66	2.9
LDL cholesterol (mmol/L)	7.3	4.1	4.1	6.3	8.6	10.4	17.4	8.2	10.5	7.4
Systolic BP at rest (mmHg)	137	105	157	151	170	132	136	105	NA	107
Diastolic BP at rest (mmHg)	108	75	91	124	132	81	107	78	NA	84
Heart rate at rest (min^-1^)	100	107	76	143	89	58	86	83	NA	94
Systolic BP during stress (mmHg)	124	88	100	134	154	129	133	78	NA	78
Diastolic BP during stress (mmHg)	93	50	44	100	116	88	105	45	NA	47
Heart rate during stress (min^-1^)	102	96	105	134	59	66	89	84	NA	96

HF = diet supplemented with 1.5% cholesterol and 15% lard, HF+c = diet supplemented with 4% cholesterol, 20% lard and 1.5% sodium cholate, BP = blood pressure

**Fig 1:**
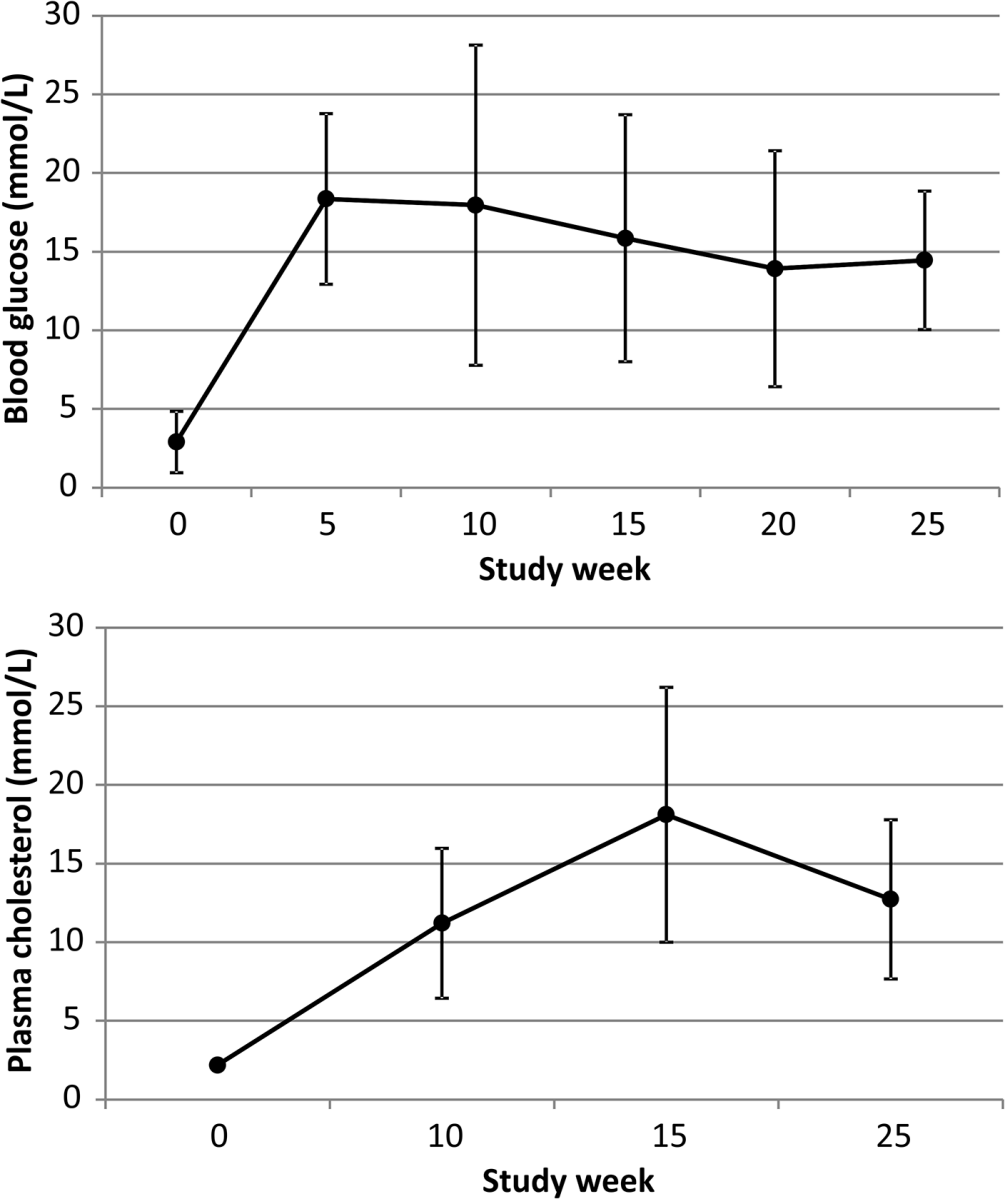
Mean fasting blood glucose and plasma cholesterol levels during 25-week follow up. The first samples (week 0) were obtained on the day of first streptozotozin injection to induce diabetes, whereas high-cholesterol diet began at week 1 and continued until the end of study.

There was an initial decrease in blood glucose levels immediately after streptozotocin injections, but then it permanently increased for the rest of follow-up (Fig 1). Plasma cholesterol level increased slowly and was at the highest level at study week 15 (Fig 1).

### Histological findings

**Fig 2:**
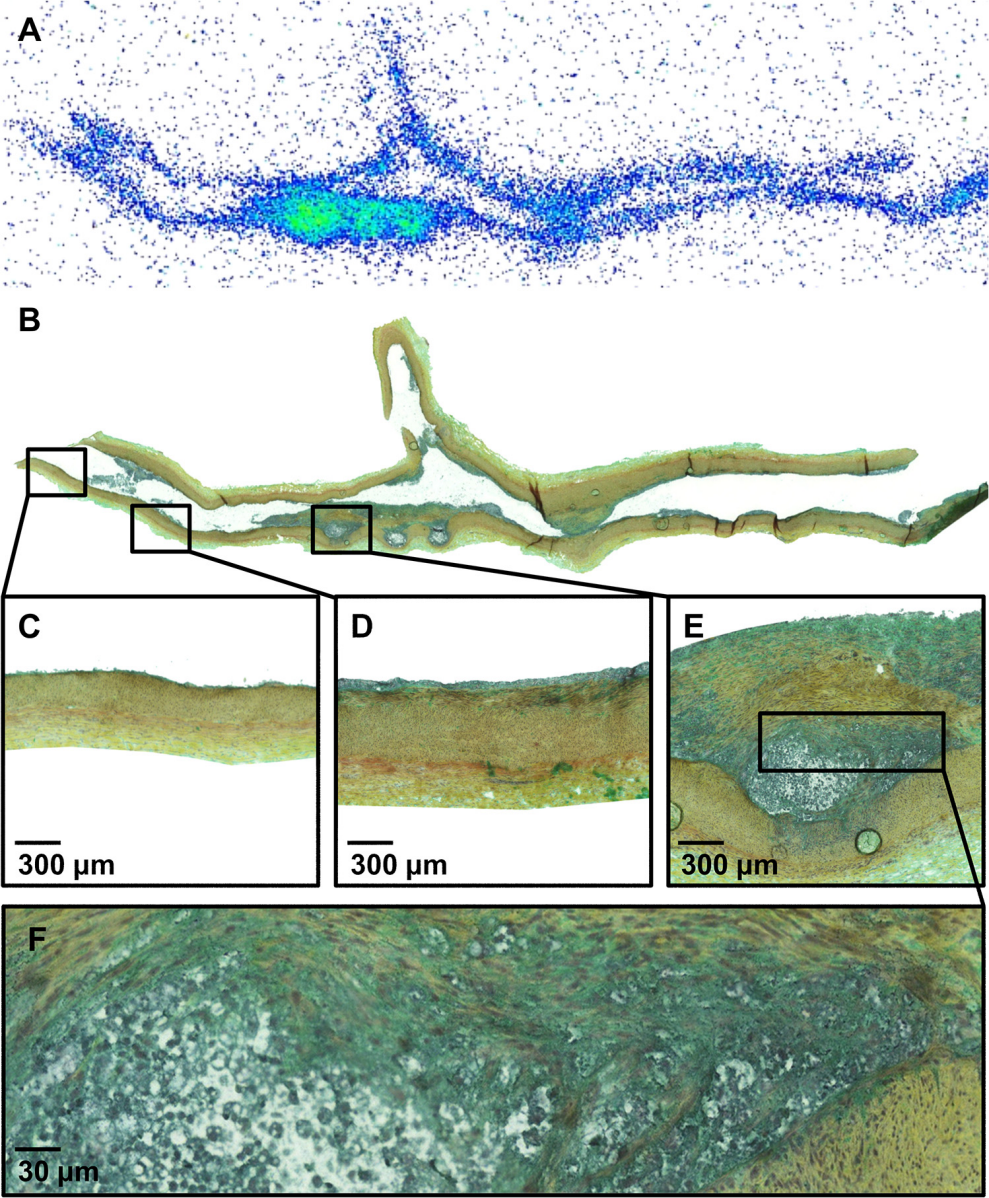
Representative [^18^F]FDG autoradiograph (A) and a serial tissue section stained with Movat pentachrome (B). The inserts show areas of normal vessel wall (C), intimal thickening (D) and atheroma (E) at higher magnification. Panel F shows lipid core surrounded by densely cellular tissue at high magnification.

A total number of 33 coronary segments (proximal LAD including the left main coronary artery = 10, mid LAD = 7, proximal LCX = 4, proximal RCA = 12) were studied. Out of these 33 segments, seven coronary segments were defined as healthy vessel wall, 16 as intimal thickening and 10 as atheroma (Fig 2). Atherosclerosis was mainly localized in the proximal part of the segments. Visual evaluation of Movat’s pentachrome stained sections showed that the lesions with intimal thickening contained high density of infiltrated cells. The atheroma lesions contained a clearly defined lipid core covered by thick layer of tissue mixed with dense cells and extracellular matrix. The average intimal cell density was 724±367 cells/mm^2^ in the areas of intimal thickening and 1059±426 cells/mm^2^ in the atheromas, *P* = 0.05. Some multinucleated inflammatory type cells were seen (Fig 2). However, macrophage immunohistochemical staining was negative. Histological findings correlated with plasma cholesterol content (R = 0.85, *P* = 0.002). Some coronary artery segments were excluded due to technical difficulties in sample collection mainly because high amount of epicardial fat.

### Autoradiography

**Fig 3:**
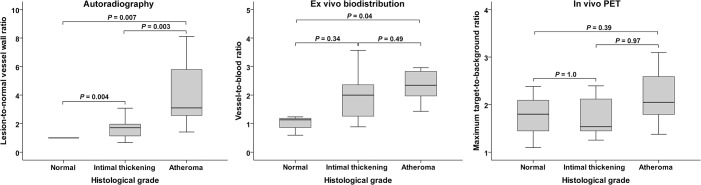
Box plots showing [^18^F]FDG accumulation in the coronary arteries by autoradiography (A), *ex vivo* biodistribution analysis (B) or *in vivo* dual-gated PET/CT imaging (C). Panel A shows [^18^F]FDG accumulation as an average plaque-to-normal vessel wall ratio as measured by autoradiography at the sites of normal vessel wall, intimal thickening or atheroma. Panel B shows average segmental [^18^F]FDG accumulation normalized to blood radioactivity in the segments without atherosclerosis, intimal thickening or atheroma plaques. Panel C shows segmental *in vivo* [^18^F]FDG signal as the highest target-to-background ratio (TBR) in the segments without atherosclerosis, intimal thickening or atheroma plaques.

Autoradiography showed focally increased accumulation of [^18^F]FDG into the coronary artery walls of diabetic and hypercholesterolemic pigs in the areas of intimal thickening (n = 16) and atheroma lesions (n = 10) as compared with the healthy vessel wall of the same animal (Fig 2). The highest lesion-to-normal vessel wall ratio was 8.1. The average uptake was 1.7±0.7 times higher in the areas of intimal thickening (*P* = 0.004) and 4.1±2.3 in the fibroatheroma plaques (*P* = 0.003) than in the adjacent, healthy vessel wall (Fig 3).

### *Ex vivo* biodistribution

The amount of [^18^F]FDG uptake in each of the 33 coronary artery segments was measured with the gamma counter and normalized to radioactivity concentration in the blood. The highest vessel-to-blood ratio of [^18^F]FDG uptake was 5.0. The average vessel-to-blood ratio of [^18^F]FDG uptake was significantly higher in the segments with atherosclerosis as compared with segments without atherosclerosis. The average vessel-to-blood ratios of [^18^F]FDG uptake were 1.3±0.7, 2.0±1.0 and 2.6±1.2 in the segments with no plaque, intimal thickening or atheroma lesions, respectively (*P* = 0.038) (Fig 3).

### *In vivo* PET/CT imaging

Due to relatively high heart rate and probably large size of the pig chest, quality of CTA images was mostly poor. This did not prevent co-localization of PET images with coronary anatomy, but was suboptimal for detailed analysis of luminal narrowing and atherosclerotic plaque. Atherosclerotic lesions seen on histology did not cause detectable luminal narrowing in the CTA images. Calcifications or detectable soft plaques were not present in the CTA images either.

**Fig 4:**
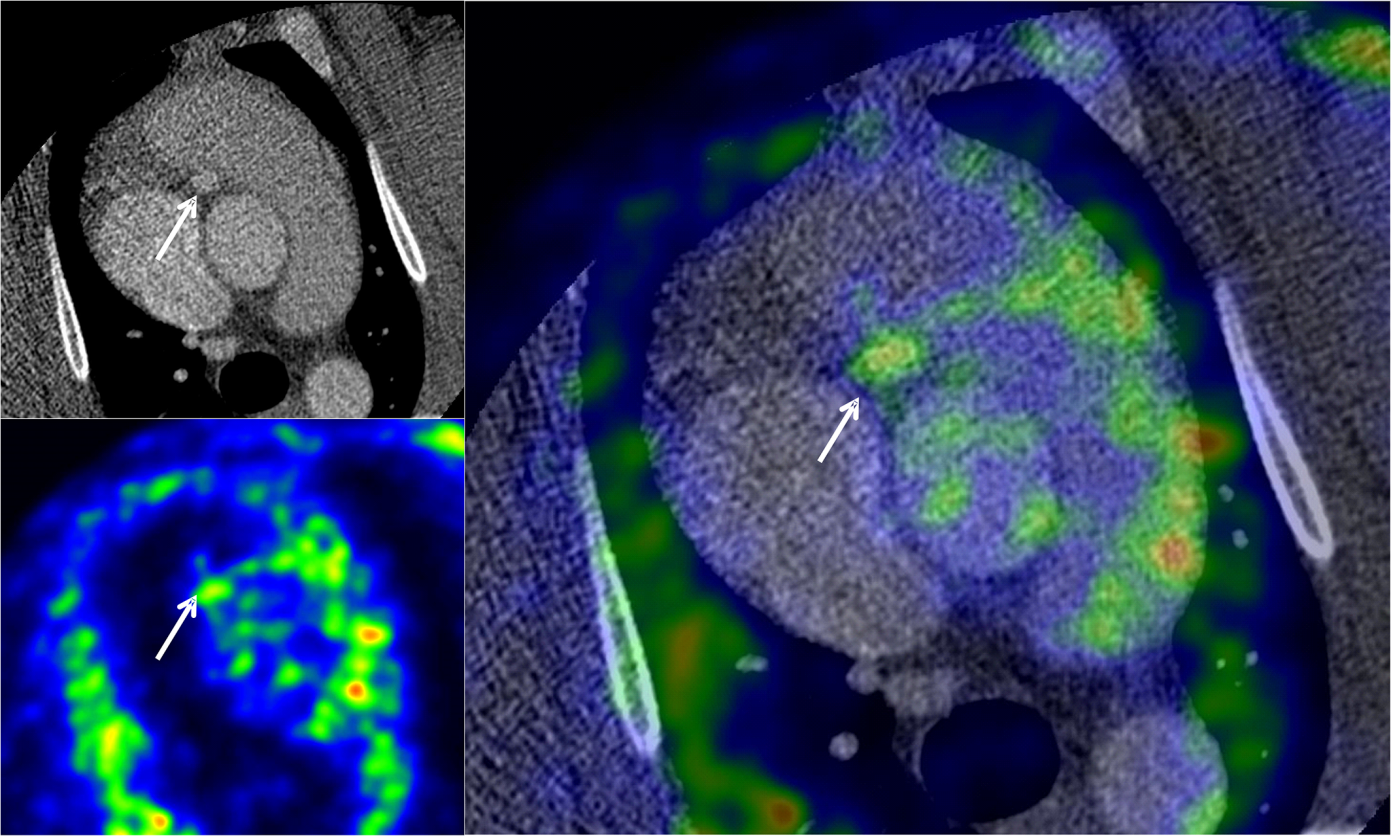
Representative example of fused axial coronary CTA and dual-gated [^18^F]FDG PET images showing cross-section of the proximal right coronary artery (arrow) in an animal with atheroma. [^18^F]FDG accumulation in the coronary arteries was measured using CTA as an anatomical reference.

Dual-gated cardiac PET images showed [^18^F]FDG accumulation above the blood pool radioactivity co-localizing with the coronary arteries as seen in the co-registered CTA image (Fig 4). In the fasting state, [^18^F]FDG uptake in the myocardium was low and areas of coronary uptake were clearly separate from myocardium. The highest target-to-background ratio, i.e. vessel uptake normalized to blood pool was 2.7 (2.0 in non-gated PET).

The peak [^18^F]FDG uptake of each coronary segment was compared with the histological finding of that segment. The average peak TBR in *in vivo* imaging was not significantly different in segments with or without atherosclerosis being 1.1±0.5, 1.2±0.4 and 1.6±0.6 in the segments with no plaque, intimal thickening and fibroatheroma, respectively (*P* = 0.4) (Fig 3).

### Microvascular function and vascular reactivity analysis

**Fig 5:**
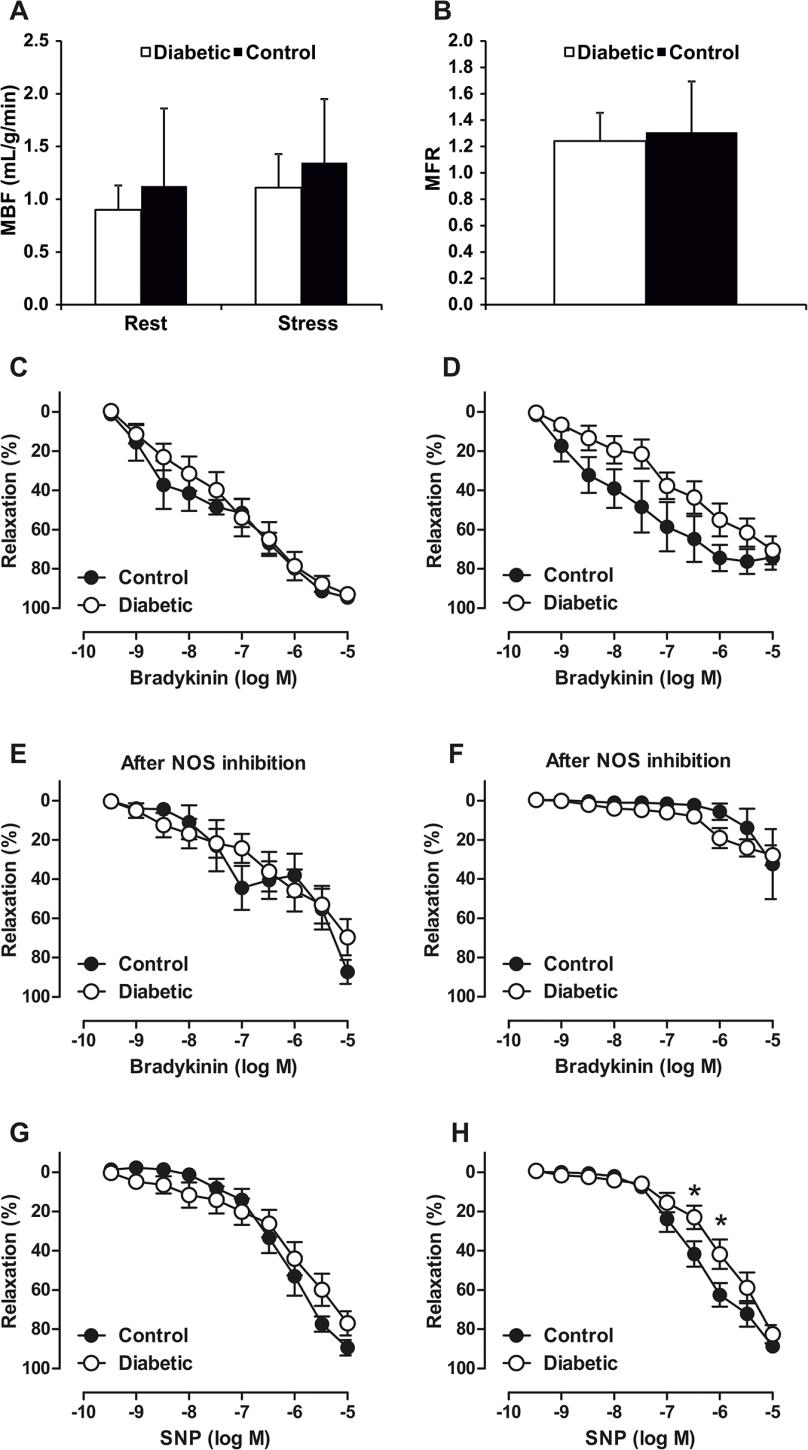
Global myocardial blood flow (MBF) at rest or stress (A) and myocardial flow reserve (MFR) (B) were comparable in diabetic (open bars) and control (black bars) animals. Wire-myograph results of distal parts of the left anterior descending artery showing different relaxation responses to bradykinin and sodium nitroprusside (SNP) of atherosclerotic and healthy animal and between small ~150 μm (C, E, G) and larger ~300 μm (D, F, H) vessel size. The contribution of vasodilator NO to bradykinin-evoked relaxation was determined by the inhibitory effect of a nitric oxide synthase (NOS) inhibitor. * *P* < 0.05 control versus diabetic at given concentration.

In order to evaluate coronary microvascular function, global MBF was quantified. MBF was comparable between diabetic and hypercholesterolemic and control animals at rest (0.90±0.23 vs. 1.12±0.74 mL/g/min, *P* = 0.88) and during adenosine stress (1.11±0.32 vs. 1.35±0.60 mL/g/min, *P* = 0.7) and MFR (1.24±0.21 vs. 1.31±0.38 mL/g/min, *P* = 0.6) (Fig 5A and 5B).

Wire-myograph measurements of distal parts of the LAD coronary arteries showed that endothelium-dependent relaxation in response to acetylcholine tended (*P* = 0.08) to be impaired in larger vessels in atherosclerotic pigs compared to healthy controls (Fig 5D). Endothelium-independent relaxation response to the NO-donor SNP was also impaired in atherosclerotic pigs, which was seen in large vessels, indicating impaired sensitivity of the vascular smooth muscle cell layer to NO-mediated vasodilatation (Fig 5H). NO synthase inhibition attenuated endothelium-dependent vasodilation more markedly in larger arteries and also abolished the initial difference in the bradykinin-evoked relaxation between healthy controls and atherosclerotic pigs. Thus, the role of NO in relaxation was higher in larger vessels revealing different relaxation mechanisms in small and large coronary arteries.

## Discussion

Increased [^18^F]FDG PET signal has been demonstrated in atherosclerotic carotid plaques and also in culprit lesions of acute coronary syndromes. The plaque development has several phases from intimal thickening to large lipid core plaque that is vulnerable to rupture. However, no data has been available to what extent [^18^F]FDG is taken up in different stages of coronary atherosclerosis. The main novel finding of this study is that [^18^F]FDG uptake is increased in coronary arteries with intimal thickening and early atheroma plaques in our pig model of atherosclerosis. To our knowledge, this is the first study where increased [^18^F]FDG uptake in different atherosclerotic coronary lesions is confirmed with histological evaluation.

The vessel wall at the site of intimal thickening and atheroma showed high cellularity. In previous studies, extensive macrophage infiltration has been observed in coronary plaques in similar model as the one used in our study [16,17]. However, probably due to species differences and technical issues associated with cryosections we did not see positive staining with antibodies against macrophage markers in the neointima and therefore, exact cell types responsible for [^18^F]FDG uptake in our study remain unknown. However, some multinucleated cells, which is a typical morphology for macrophages were seen. However, previous studies have shown that [^18^F]FDG signal closely correlates with macrophages with high rate of glucose metabolism [3]. Macrophages are good target for molecular imaging of atherosclerotic plaque inflammation not only because their high metabolic activity but also presence in very early phase of the disease [24]. Our findings indicate that increased metabolic activity detected by [^18^F]FDG is a typical feature of even early coronary atherosclerosis.

The second aim of our study was to explore the feasibility of detecting [^18^F]FDG uptake in coronary atherosclerotic lesions by *in vivo* PET/CT. Increased accumulation of [^18^F]FDG was successfully visualised in atherosclerotic lesions with autoradiography and biodistribution study. However, no differences of [^18^F]FDG accumulation were seen between healthy vessel wall, intimal thickening and fibroatheroma by *in vivo* PET. We used dual-gated PET method that has shown better vessel-to-blood ratio values than the non-gated PET [6]. In the present study the highest TBR in dual-gated PET was 2.7 and 2.0 in non-gated PET. It appears, however, that sensitivity of *in vivo* PET is still too low to detect differences in the [^18^F]FDG coronary accumulation between healthy vessel, intimal thickening and fibroatheroma in small atherosclerotic lesions as seen in our study. Physiological uptake of [^18^F]FDG in the myocardium was efficiently blocked by high-fat diet and fasting of animals overnight before the study. The protocol was similar to pre-treatment successfully used to reduce myocardial [^18^F]FDG uptake previously in human studies [18]. Our results indirectly support the previous findings that [^18^F]FDG uptake in the coronary arteries visible in PET images represent later stages of atherosclerosis with more intense inflammation, such as in the culprit lesions of acute coronary syndromes [9,11]. Further studies in models with more advanced atherosclerosis are needed to explore the actual morphological correlates of coronary [^18^F]FDG uptake.

The atherosclerotic changes were induced in a pig model already after 6 months by combination of diabetes and hypercholesterolemia. The finding of coronary atherosclerosis is consistent with earlier studies using similar model [16,17]. However, unlike in previous studies more advanced lesions were not present in our study despite similar study duration, degree of hyperglycemia and hypercholesterolemia. The lack of advanced lesions may be explained by different responses between pigs of different genetic background (Yorkshire in previous and Finnish landrace in our study). In our model, supplemental sodium cholate significantly accelerated atherosclerosis as shown by more atheroma lesions in pigs with than without cholate supplement. Significant coronary stenosis was not detected by either invasive coronary CTA or histology. Consistent with this there were no regional abnormalities in myocardial perfusion during adenosine stress. Previous studies indicate that mechanical constrictors or stents can be used to induce regional ischemia [25,26]. Although *in vivo* PET imaging and coronary CTA were feasible in this model, our results indicate that models of either more aggressive atherosclerosis or prolonged exposure to diabetes and hypercholesterolemia would be needed to induce haemodynamically significant coronary stenosis in pigs.

MFR was low in pigs as compared with humans. However, MFR was comparable in atherosclerotic and age matched, healthy pigs indicating a species or protocol related phenomenon rather than disease related change. Despite the absence of microvascular dysfunction evaluated with MBF quantification, further vascular reactivity analysis showed impairment in relaxation responses revealing possible endothelial dysfunction also in distal vessel segments. These findings indicate that short term diabetes and hypercholesterolemia as such can induce impairment of endothelial function.

There are some obvious limitations in our study. The pig atherosclerotic lesions induced by short-term diet may not correspond to the morphology of the advanced human coronary atherosclerotic plaques developed over years. Furthermore, we were not able to define exact cell types in the neointima in our model. However, more prolonged period of diabetes and hypercholesterolemia was not possible in our study, because of rapid growth of the animals that would have made imaging studies impossible. [^18^F]FDG imaging was not performed in a healthy control group, because high blood glucose levels in diabetic animals could have made comparisons unreliable. It has been shown that even moderate hyperglycaemia can lower [^18^F]FDG uptake by plaque cells. This is probably mainly based on competition between glucose and [^18^F]FDG as metabolic substrates [27]. Instead of control group, healthy coronary segments from the same animals were used as a reference. Finally, we were not able to compare [^18^F]FDG uptake into atherosclerotic lesions in different vascular beds, because there was no atherosclerosis in the aortas of the pigs and carotid vessels were prepared for injection of tracers and obtaining blood samples.

## Conclusions

In early coronary atherosclerotic lesions in a pig model of diabetes and hypercholesterolemia plaque inflammation and clearly increased uptake of [^18^F]FDG can be detected. However, this uptake was not detectable by *in vivo* imaging in these lesions representing intimal thickening or small atheromas. Further studies are needed to define the determinants of [^18^F]FDG PET signal in more advanced coronary atherosclerotic lesions.

## Supporting Information

S1 Dataset Minimal dataset. (XLS)
